# Building a collaboration model between the primary and secondary mental healthcare levels to improve care for trauma-exposed children in Norway: study protocol for the NorStep Collaborate study

**DOI:** 10.1136/bmjopen-2026-120162

**Published:** 2026-07-10

**Authors:** Silje M Ormhaug, Pernille Gurandsrud, Kristin Johanne Økern Haabrekke, Guro Haga, Irene Wormdahl, Marie Lindebø Knutsen

**Affiliations:** 1 Norwegian Centre for Violence and Traumatic Stress Studies, Oslo, Norway; 2 Diversity and Inclusion, NTNU Samfunnsforskning AS, Trondheim, Norway

**Keywords:** MENTAL HEALTH, Child & adolescent psychiatry, Organisation of health services

## Abstract

**Abstract:**

**Introduction:**

Childhood trauma is a public health challenge, and low-threshold interventions are important to prevent long-term problems. A stepped care approach is recommended so the intensity of care can be adjusted according to the child’s needs. This approach requires coordination and collaboration between service levels. However, interagency collaboration can be challenging. The aim of the NorStep Collaborate study is to develop and evaluate a model for collaboration between the primary and secondary mental healthcare levels in Norway.

**Methods and analysis:**

NorStep Collaborate is part of an ongoing randomised controlled trial investigating the effectiveness of a low-threshold, parent-led trauma treatment provided in the primary care level services. Children in need of more therapist-intensive treatment will be stepped up to the secondary care level. NorStep Collaborate is based on principles of participatory action research, and the collaboration model will be developed together with stakeholders from six primary level services and four secondary level services. In phase I, the model will be developed. Stakeholders will participate in focus group interviews and full-day dialogue conferences to suggest and discuss routines for collaboration and a suggested collaboration model will be refined through feedback meetings. In phases II and III, the co-created model will be implemented and tested for 1 year, and therapists, leaders, children and parents will be interviewed about their experiences with the collaboration and transfer between the primary and secondary care level. Interviews will be analysed with thematic analyses. In phase IV, results of the evaluation will be shared, and final adjustments made if necessary.

**Ethics and dissemination:**

The study has been evaluated by the Norwegian Agency for Shared Services in Education and Research (SIKT, reference number 161459) and is approved by the data protection officer at Norwegian Centre for Violence and Traumatic Stress Studies, Norway. Participants will be provided with written information about the study, and participation is voluntary, based on verbal consent and assent. The findings will be presented to the Norwegian Ministry of Health and Care, all participating services and the professional public via conferences, and in international and Norwegian peer-reviewed publications.

**STRENGTHS AND LIMITATIONS OF THIS STUDY**

- A strength of this study is that the collaboration model that will be developed is based on stakeholders’ own perceived needs and solutions, and will be implemented, evaluated and adjusted as needed.
- The collaboration model will be evaluated both in terms of quality of participation (stakeholder involvement and to what degree the proposed actions were actually implemented) and outcome of the model (what are therapists’, leaders’, parents’ and children’s experiences with the collaboration after the model has been implemented).
- Due to limitations of stakeholders’ time and resources, stakeholders will not be included as equal partners in the analysing phases, but an iterative strategy will be used to ensure stakeholder feedback during the whole process of development and evaluation of the model.
- This study does not include a randomised control condition, so conclusions on the effectiveness of the collaboration model cannot be drawn.

## Introduction

 Every year, a substantial number of children and adolescents in Norway are exposed to various forms of potentially traumatic events, violence and abuse.^1 2^ These are events that present a high risk of developing severe health problems^3 4^ that can lead to lifelong consequences if left untreated.^3 5^ Studies have shown that trauma-exposed children have a higher likelihood of dropping out of school and education and are more likely to disengage from the workforce compared with their non-exposed peers.^6^ These outcomes not only affect individual well-being but also generate significant economic and societal costs.^7^ Childhood trauma is thus a major public health challenge,^5 8^ and the provision of accessible, low-threshold interventions for affected children and youth is important to prevent initial symptoms from developing into more severe problems.

Currently, there is a gap between the need for and access to trauma-informed care.^9 10^ One measure to increase access to treatment is to adjust the intensity of care to the children’s needs with a stepped care approach.^11^ The model Stepped Care Cognitive Behavioural Therapy for Children after Trauma (SC-CBT-CT)^12 13^ is a promising and cost-effective intervention for trauma-exposed children. Step 1 of the model, Stepping Together for Children after Trauma (ST-CT), is parent-led, and the responsibility to provide the treatment is partially shifted to the parents under the close supervision of a therapist. For children who do not achieve sufficient improvement after step 1, step 2 comprises standard therapist-led trauma-focused CBT (TF-CBT^14^). So far, studies show that SC-CBT-CT is an effective model, where 2/3 of the children respond to step 1, and costs are approximately 50% lower compared with standard TF-CBT.^13^ In the original model, both steps are provided by the same therapists, but in Norway, the Norwegian Centre for Violence and Traumatic Stress Studies (NKVTS) has tested an adapted version where Step 1 of the intervention is provided by the primary care level services and step 2 is provided by the secondary care level’s child and adolescent mental health clinics (CAMHS). This adaptation was made since the primary care level services in Norway are more accessible, have less wait-time, and therefore can provide treatment at an earlier time point. Results from an initial feasibility study show that the parent-led step 1 is both feasible and acceptable in a Norwegian primary service level context, and that the response rate is comparable to the original model.^15^ Based on these findings, NKVTS initiated a randomised controlled trial (RCT)—The NorStep RCT study—to evaluate the effectiveness of the step 1 ST-CT compared with treatment as usual in primary care level services (for more details, see Ormhaug *et al*^16^ and trial registration ClinicalTrials.gov Identifier NCT04073862). Drawing on findings from the feasibility study, it is anticipated that approximately 20%–30% of children receiving the parent-led ST-CT intervention will be stepped up to receive further treatment in the secondary level CAMHS. However, interviews with families from the feasibility study showed that the transfer from the primary to secondary care level was not ideal.^17^ For children exposed to trauma, maximum wait-time is set to 8 weeks^18^ and many parents experienced this wait time to be too long and difficult. Also, the TF-CBT therapists at the CAMHS described that they did not know enough about the step 1 ST-CT treatment to be able to tailor the TF-CBT intervention in a way that built on the work that had already been done. As a result, the interventions provided appeared uncoordinated and less helpful for the families. These findings are in line with other studies investigating both parents’ and therapists’ experiences of collaboration between welfare professionals and across service levels.^19 20^

In Norway, interservice collaboration between primary and secondary healthcare services for children and adolescents is legally mandated. The obligation to collaborate is anchored in both the Health and Care Services Act and the Specialist Health Services Act, which require the health service levels to establish structures that facilitate cooperation. For children and adolescents with mental health problems, this framework is intended to secure integrated health service across levels and sectors. In support of this goal, a meta-analysis found that when services collaborated in line with integrated care-models, this was less costly and patients reported higher levels of health-related quality of life compared with patients receiving ordinary care.^21^ However, interorganisational collaboration can be challenging as it often requires a willingness to change current routines and engage in ‘risky behaviours’, such as investing time and resources in performing tasks intended to increase collaboration or trusting that collaborating partners are able to provide good enough services.^22^ In line with this, mutual trust and faith in the collaboration process have been identified as two of the most central mechanisms for a successful collaboration, whereas a history of poor collaboration, differences in regulations, lack of common communication systems and staff turnover are examples of mechanisms that can impede collaboration.^22 23^ Measures to increase trust and faith in collaboration include actively involving stakeholders in the development of collaborative models, developing a shared vision of the goals and obstacles for collaboration, securing leadership involvement and installing a sense of shared responsibility and accountability in both parties.^22^

In sum, effective services and well-established collaboration practices are needed to ensure better care for traumatised children and to aid both the primary mental health services and CAMHS in fulfilling their responsibilities. The aim of the NorStep Collaborate study is to involve stakeholders to develop, implement and evaluate a feasible and effective model for collaboration between primary and secondary healthcare levels, specifically to improve the transfer between service levels for traumatised children who are stepped up from ST-CT to standard TF-CBT.

## Research questions

1. What actions should a collaboration model between the primary and secondary mental healthcare levels include?
2. What are the barriers and facilitators of implementing the new collaboration model?
3. How do therapists, leaders, children and parents perceive the transition from the primary to the secondary care level when the new collaboration model is implemented?

## Methods and analysis

### Study design

The study has an exploratory, qualitative design, building on the principles of participatory action research. Participatory action research is a collaborative and action-oriented approach that involves researchers and community members working together to identify problems, generate knowledge and implement solutions.^24 25^ The study is inspired by the Generative Co-Design Framework for Healthcare Innovation.^26^ Based on the summary by Vargas *et al*^27^ the principles guiding the co-design process can be defined as participative (we will include collaborative workshops), iterative (feedback loops will help refine and improve solutions), supportive (the project group will take care of all practicalities involved in the development process), applicable (the model will be tested for feasibility and utility), and transparent (participating services will receive open communication about the process). The collaborative workshops will be arranged according to the model for dialogue conferences,^28^ which emphasise principles of democratic participation and equality among stakeholders, dialogical interaction, shared decision-making and ownership to facilitate stakeholder-driven changes in practice. Overall, the study will follow the steps of the expansive learning cycle^29^ and is divided into four phases. In phase I, the model for collaboration between primary and secondary mental healthcare services will be developed. This will happen through a co-creation process with four pilot teams, each team consisting of therapists and leaders from locally collaborating healthcare services from both service levels. In phase II, the models will be implemented. This includes formalising the new routines, informing all parties involved and adapting the clinical practice according to the new routines. This phase will last for 1 year. Along with the implementation, in phase III, regular check-ins (to provide implementation support) and interviews with therapists and leaders will be conducted, as well as interviews with parents and children who are ‘stepped up’ during the trial period in order to evaluate the model. At the end of the year, therapists and leaders will also be invited to share their experiences. In the final phase IV, results from the interviews will be summarised and presented to the pilot-teams for feedback. Based on the results, the model for collaboration will either be consolidated or, if needed, adjusted and re-tested.

### Study setting

Norwegian health services are publicly funded and of no cost for the families. The healthcare services for children are provided at three levels. The municipalities are responsible for the primary care level, with the responsibility to treat mild to moderate mental health symptoms. The services are intended to be easily accessible, and parents and families can contact the services directly without referral from a third party (eg, general practitioners or child protective services). When symptoms are considered moderate to severe, children will be referred to the secondary care level to receive help at the outpatient CAMHS or to the third level for in-patient treatment. The CAMHS and in-patient clinics are the responsibility of the state, and children and families need a referral from either their general practitioner, the child protective services or a psychologist from the primary care level to be admitted.

### Participants and recruitment

The services and stakeholders participating in the NorStep Collaborate study have been recruited from the primary care services participating in the ongoing NorStep RCT study. Thirteen of the 21 services that participated in the RCT study in Fall 2024 were invited, based on their size (the largest services were selected), geography (services that were geographically and administratively situated in proximity to other services in the study were prioritised) and characteristics of their corresponding CAMHS (they were required to provide TF-CBT). Of the 13 services contacted, six services agreed to participate, together with four associated CAMHS at the secondary care level. This resulted in four NorStep Collaborate pilot teams, of which two of the teams consist of one primary care service and their corresponding CAMHS, and two teams consisting of two primary care services and each of their corresponding CAMHS. The current study thus includes 10 services in total. For stakeholder participation, the study researchers will encourage team leaders and therapists trained in either ST-CT or TF-CBT to participate in the project, but all participation is voluntary and decided by the therapists and leaders themselves.

The study will also include children and caregivers with experience of being stepped up from the primary to the secondary care level. These children and caregivers will be recruited among participants in the NorStep RCT study. First, families with experiences of being stepped up before the collaboration model is developed will be interviewed, in order to gain an understanding of their needs and experiences with the transfer (see phase I below). Participation in the interviews will be based on verbal consent from caregivers (on behalf of both themselves and their child), and verbal assent from the children. second, after the collaboration model has been developed and is under evaluation (see phases II and III below), children and caregivers from the six primary care services involved in the NorStep Collaborate study will be invited to interviews. These interviews will provide perspectives of children and families with experience from collaboration between primary and secondary care services and can contribute to a better understanding of the outcome of the collaboration model in terms of client satisfaction.

To ensure sufficient involvement from the stakeholders, the Research Council of Norway (NFR) requires that at least 10% of the budget is allocated to the collaborating partners. Therefore, each of the 10 participating services will be provided with financial support of 100 000 NOK, distributed over the 3 years the study will take place.

The study will be conducted between Spring of 2025 and Spring of 2028.

### Phase I: building the model through co-creation

To prepare for the co-creation process, focus group interviews will be conducted with each participating service through digital video meetings. All therapists (minimum 2 TF-CBT/ST-CT therapist+their colleagues) and their team leaders (n=4) will be invited to participate, comprising between 12–60 participants in total. We will prepare open questions, asking for their existing routines for collaboration, both in general and for trauma-exposed children specifically. They will also be asked about what their ideal collaboration with their associated primary or secondary healthcare service looks like. Participation is voluntary and the digital video meetings will be recorded based on verbal consent from the participants. After the completion, the interviews will be transcribed and analysed by the research group, and common themes and experiences from the different services will be defined. Further, children and caregivers from the NorStep RCT study who have been stepped up to the secondary care level will be contacted and interviewed with semi-structured interviews about their experiences with the transfer between services, what they think worked well and what they wish would have been different. Participation in the interviews will be based on verbal consent from caregivers (on behalf of both themselves and their child), and verbal assent from the children. We expect that approximately five to eight families will be eligible for interviews, based on our experience with the rate of children that are stepped up in the RCT study so far. Together, these interviews will give us a better understanding of the current routines and level of collaboration, what they miss, and what should be improved from the view of both service providers and users.

The co-creation process will follow the design of the Norwegian ReCoN study.^30^ Following the focus group interviews, four full-day dialogue conferences will be arranged, one with each pilot team. The dialogue conferences will consist of a short theoretical lecture with information and aims of the project and the conference, a brief introduction to the SC-CBT-CT model, and a summary of the findings from the focus group interviews. The main part of the day will consist of group work sessions. In the first session, participants will sit together with colleagues from their own service and will be asked to brainstorm and come up with suggested actions hypothesised to improve the collaboration. In the second session, groups will be mixed across services with the task to review all the suggested actions and prioritise the seven actions they find most important and, at the same time, feasible to implement within their current work context. These prioritised actions from each group will be collected and then presented and displayed on posters around the room. Finally, each participant will be given three stickers and will be asked to indicate which actions they find most important by placing their stickers on the corresponding measures. Throughout the day there will be multiple coffee breaks and a long lunch to facilitate relationship building among stakeholders in the different services, and all practicalities regarding the arrangement of the conferences will be handled by the research group.

After completion of the four dialogue conferences, the researchers will perform inductive thematic analysis^31^ to interpret and systematise the contributions from the dialogue conferences. This analytical process will produce a proposed collaboration model that can be feasible across the pilot teams. A draft of the model will be sent to each participating service for review and feedback to prepare for a final meeting. Here, two representatives from each of the services included in this study will participate and agree on a collaboration model that seems both feasible and effective. See table 1 for a timeline of phase I.

**Table 1:** Timeline phase I: co-creation process

Months	Activity	Participants
0–1	Digital kick-off meetings	Leaders and all team members, each service separately
2-3	Focus group interviews	Leaders and team members, each service separately
6–8	Dialogue conferences	Leaders and team members from primary and secondary care services together, each pilot-team separately
10	Feedback first draft of model	Written feedback
11	Finalising meeting	Leaders and one therapist from each participating service together
12	Feedback final model	Written feedback from services to finalise model

### Phase II and III: implementation and evaluation of the model

After development, the next phases consist of a 1-year parallel implementation (phase II) and evaluation of the collaboration model (phase III). Phase II will start with a kick-off meeting where the model is presented to all team members of the participating services. In phase III, the level of implementation and perceived effect of the model will be monitored and evaluated. The evaluation will consist of bi-monthly check-ins to provide implementation support and hear whether the decided measures have been implemented in order to evaluate quality of participation and four semistructured interviews with participating therapists and leaders to investigate potential barriers and facilitators for implementation, in addition to their perceived outcome of the model.

Children and caregivers who are stepped up from primary to secondary care levels during the test year will be interviewed about how they perceive this transition under the new framework. Participation in the interviews will be based on verbal consent from caregivers (on behalf of both themselves and their child) and verbal assent from the children. Interviews will be conducted between 6 and 8 weeks after referral to the CAMHS. Again, it is expected that around five to eight families will be eligible for interviews during phase III.

In total, we anticipate performing around 80 individual interviews with professional participants and 10–16 interviews with children and caregivers. See table 2 for an overview of the planned interviews in the study.

**Table 2:** Overview of interviews and implementation support in phases I–III

RQ	Phase, timing	Topic of interest	Questions to be addressed in interviews	Type of interview	Participants (expected n)
1	I	Baseline*:* Current status of collaboration	Current routines for collaboration Perceived quality of collaboration What will the ideal form of collaboration look like	Focus group, open questions	Leaders, therapists (between 12 and 60)
1	I	Baseline*:* Experiences with transfer between service levels before collaboration model	Experiences with transfer, including wait-time and change of therapist, perceived continuity of care across levels Suggested improvements in collaboration	Semistructured, individual	Children, caregivers (ca 5)
2	II Month 1	Baseline: Expectancies for collaboration model	Expectations of how well both own and collaborating services will implement actions in collaboration model Expectations on outcome of collaboration model	Semistructured, individual	Leaders, therapists (ca 20)
2	II Months 2, 4, 6, 8, 10	Quality: Level of implementation	Checklist of actions included in collaboration model Barriers and facilitators for implementation of actions	Structured interview, individual	Leaders, therapists (ca 10)
3	III Months 4, 6, 12	Outcome: Experiences with collaboration model	Perceived changes in collaboration Perceived facilitators and barriers	Semistructured, individual	Leaders, therapists (ca 20)
3	III 4–6 weeks after transfer	Outcome: Experiences with transfer between service levels after collaboration model	Experiences with transfer, including wait-time and change of therapist Perceived continuity of care across levels	Semistructured, individual	Children, caregivers (ca 5)

RQ, research question.

All interviews will be conducted over video and recorded with end-to-end encryption, transcribed and stored in a secure server managed by Services for Sensitive Data (TSD) at the University of Oslo.

### Phase IV: analysis, summary of evaluation and final consolidation

In phase IV, the interview data from phase III will be analysed. We will apply a reflexive thematic analysis of the data^31^ to evaluate implementation progress and level, identify specific barriers and facilitators to implementation, and explore the participants’ experiences with the model. All authors will take part in the analysis.

Finally, the results from the interviews will be summarised and presented to the pilot teams for their feedback. Based on the results, the collaboration model will be adjusted and consolidated.

### Overview of interviews

The study will include several interviews to collect information both before, during and after the implementation of the collaboration model. See table 2 for an overview.

### Patient and public involvement

The idea and research questions for this study are based on feedback from therapists and families participating in the NorStep feasibility study.^15^ Stakeholders (leaders and therapists) and children and caregivers with experience of being stepped up will be involved in this study from phase I. Leaders and therapists will take part in focus groups, dialogue conferences and iterative feedback loops on model drafts to co-design and refine the collaboration model. They will also be asked about the perceived burden of participating in the project. Children and caregivers who have been stepped up from primary to secondary care in the NorStep RCT will be interviewed about their experiences of this transition, and their priorities and perceived needs will inform the development of an effective model for collaboration between primary and secondary healthcare levels. The results/evaluation will be fed back to the pilot teams to finalise the model. The authors will disseminate the results in peer-reviewed articles, presentations at academic conferences, and in meetings and reports to the Norwegian Directorate of Health and Care, with the perspectives from stakeholders, children and families participating in the study reflected in the final recommendations.

## Discussion

In this study, a new collaboration model between the primary and secondary mental health service levels will be developed. In Norway, collaboration between primary care health services and secondary health services for children and adolescents is a statutory obligation (the Health and Care Services Act, the Specialist Health Services Act). The aim is to ensure coordinated and coherent services for children and families across levels of care. However, as learnt from our pilot study,^17^ this can be challenging to achieve. Results from the current study may provide important knowledge to facilitate collaboration and improve transitions between the primary and secondary mental healthcare levels. Although this study focuses on care for trauma-exposed children, improved and more effective routines for collaboration across service levels are likely to benefit all children in need of coordinated mental healthcare. A key strength of this study is the co-creation process and the active involvement of stakeholders in the development of the collaboration model, which is expected to enhance the model’s acceptability and feasibility.^22^

This study has some limitations. First, the development process is relatively time-consuming, as it involves multiple steps and several stakeholders. However, this process may be an important investment in building stakeholders’ trust and faith in the final model,^22^ as well as increasing the proposed model’s feasibility for users and services and, thus, research’s societal impact.^25 32^ Second, to increase feasibility of the study, stakeholders’ involvement has been restricted to include input on current status of collaboration, suggestions of actions in new collaboration model, feedback on suggested model, and perceived experiences with model after implementation. Stakeholders will not be involved in analyses of interviews of suggested content. Third, the lack of a control condition makes it difficult to isolate the effect of the new collaboration model. Conclusions about the effect will be drawn based on participants’ subjective impressions, and these can be biased due to expectancy effect or a desire to favourably assess ‘their own’ model as successful.

Future studies should include more services (child protective services, school nurses, etc) to learn how a more complex network of services can collaborate and a comparison group to be able to test the effect of a collaboration model.

To conclude, in Norway, the primary and secondary service levels are formally mandated to collaborate, and the stakeholders are calling for improvements in collaboration.^17^ Given that some trauma-exposed children require follow-up from both primary and secondary health service levels, there is a clear need for more knowledge on how collaboration between the levels can be improved. Therefore, participatory research, where the stakeholders themselves take part in developing a model for collaboration, co-created and tailored to their everyday work context, appears to be a promising approach for generating a relevant and practice-oriented collaboration model, and, most importantly, to improve integrated treatment and help for children and families in need of coordinated care from the two service levels.

### Ethics and dissemination

The study has been evaluated and approved by the Norwegian Agency for Shared Services in Education and Research (SIKT, reference number 161459) and the data protection officer at NKVTS, Norway.

All participation in the study during all the phases is voluntary and will be based on verbal consent and assent. Participants will receive written information about the aim of the study, what it will require to participate, how data will be handled, and that they can withdraw at any time point. Recordings from the interviews will be stored in a secure server managed by TSD at the University of Oslo.

The results from this study will be submitted for publication in international and Norwegian peer-reviewed journals. In addition, the findings will be disseminated to the Norwegian Ministry of Health and Care and the participating health services, as well as presented at relevant research and clinical conferences.
