# Supplementary material for: Building a collaboration model between the primary and secondary mental healthcare levels to improve care for trauma-exposed children in Norway: study protocol for the NorStep Collaborate study
Source: BMJ Open. 2026 Jul 10;16(7):e120162. doi: 10.1136/bmjopen-2026-120162 (PMC13358233; doi:10.1136/bmjopen-2026-120162)
Supplement: Supplementary file 1 [file bmjopen-2026-120162.reviewer_comments.pdf]

PEER REVIEW HISTORY

BMJ Open publishes all reviews undertaken for accepted manuscripts. Reviewers are asked to complete a checklist review form and are provided with free text boxes to elaborate on their assessment. These free text comments are reproduced below.

ARTICLE DETAILS

Title (Provisional)

Building a Collaboration Model Between the Primary and Secondary Mental Health Care Levels to Improve Care for Trauma-Exposed Children: Study Protocol for the NorStep Collaborate Study

Authors

Ormhaug, Silje M.; Gurandsrud, Pernille; Haabrekke, Kristin Johanne Økern; Haga, Guro; Wormdahl, Irene; Lindebø Knutsen, Marie

---

VERSION 1 - REVIEW

**Reviewer**	**1**
**Name**	**Zeng, Irene**
**Affiliation**	**Auckland University of Technology Faculty of Health and Environmental Sciences, Health Falculty research office**
**Date**	**18-May-2026**
**COI**	**None**

I would like to extend my congratulations to all the authors for completing a very important and innovative study, co-design and evaluate a primary-secondary integrated care TB interventional program.

The manuscript is well written; I have a few recommendations as follows:

1. What framework was applied in the study and what principles will be applied in the co-design process? For example, democracy, empowerment, and shared decision-making, need to be describe in the method.

2. Important references for co-design (participatory or collaborative) principles should be included, here are some typical suggestions:

*Robert G, Locock L, Williams O, Cornwell J, Donetto S, Goodrich J. Co-Producing and Co-Designing. Cambridge University Press; 2022.

*Woodward M, Dixon-Woods M, Randall W, et alHow to co-design a prototype of a clinical practice tool: a framework with practical guidance and a case study BMJ Quality & Safety 2024;33:258-270.

*Morley C, Jose K, Hall SE, et alEvidence-informed, experience-based co-design: a novel framework integrating research evidence and lived experience in priority-setting and co-design of health servicesBMJ Open 2024;14:e084620.

*Carmen Vargas, Christina Zorbas, Giuliana Raffaella Longworth, Anna Ugalde, Cindy Needham, Athira Sunil, Carolina Venegas Hargous, Rebecca Bennett, Tari Forrester-Bowling, Ana Paula Cardoso Richter, Steven Allender, Exploring co-design: a systematic review of concepts, processes, models, and frameworks used in public health research, Journal of Public Health, Volume 47, Issue 4, December 2025, Pages e616–e639, https://doi.org/10.1093/pubmed/fdaf084

*Pelton, L., and M. Knihtila. "Experience-Based Co-Design of health care services implementation guide." Institute for Healthcare Improvement, Boston, Massachusetts (2018).

3. Co-design/process for implementation from synthesising guidelines are recommended as follows:

• Engage stakeholders early (problem definition stage)

• Be explicit about roles and decision-making power

• Use multiple participatory methods

• Ensure ongoing iteration and feedback loops

• Invest in facilitation and relationship-building

• Evaluate both:

o outcomes

o quality of participation

4. In the current protocol, the outcomes have not defined for children outcomes and quality of participation.

Other minor comments:

1. The co-design include interview; it is not specified the interview method is semi-structure or in depth personal. Interview method needs to be described and justified.

2. The title include evaluation, while the method has not mentioned the outcomes or future plan for evaluation in the co-designed model. Children's TB outcomes are not included in the evaluation.

VERSION 1 - AUTHOR RESPONSE

Reviewer: 1
Dr. Irene Zeng, Auckland University of Technology Faculty of Health and Environmental Sciences

Comments to the Author:
I would like to extend my congratulations to all the authors for completing a very important and innovative study, co-design and evaluate a primary-secondary integrated care TB interventional program.
The manuscript is well written

Response: Dear Dr. Zeng, we very much appreciate your thorough reading and all your helpful comments!

I have a few recommendations as follows:

1. What framework was applied in the study and what principles will be applied in the co-design process? For example, democracy, empowerment, and shared decision-making, need to be describe in the method.
Response: Thank you for this comment. A description of the framework and principles that will be applied in the co-design process is added in the method section (p.6).

Page 6: *The study is inspired by the Generative Co-Design Framework for Healthcare Innovation (27). Based on the summary by Vargas et al. (28) the principles guiding the co-design process can be defined as participative (we will include collaborative workshops), iterative (feedback loops will help refine and improve solutions), supportive (the project group will take care of all practicalities involved in the development process), applicable (the model will be tested for feasibility and utility), and transparent (participating services will receive open communication about the process). The collaborative workshops will be arranged according to the model for dialogue conferences (29), which emphasise principles of democratic participation and equality among stakeholders, dialogical interaction, shared decision-making and ownership to facilitate stakeholder-driven changes in practice.*

2. Important references for co-design (participatory or collaborative) principles should be included, here are some typical suggestions:
*Robert G, Locock L, Williams O, Cornwell J, Donetto S, Goodrich J. Co-Producing and Co-Designing. Cambridge University Press; 2022.
*Woodward M, Dixon-Woods M, Randall W, et alHow to co-design a prototype of a clinical practice tool: a framework with practical guidance and a case study BMJ Quality & Safety 2024;33:258-270.
*Morley C, Jose K, Hall SE, et alEvidence-informed, experience-based co-design: a novel framework integrating research evidence and lived experience in priority-setting and co-design of health servicesBMJ Open 2024;14:e084620.
*Carmen Vargas, Christina Zorbas, Giuliana Raffaella Longworth, Anna Ugalde, Cindy Needham, Athira Sunil, Carolina Venegas Hargous, Rebecca Bennett, Tari Forrester-Bowling, Ana Paula Cardoso Richter, Steven Allender, Exploring co-design: a systematic review of concepts, processes, models, and frameworks used in public health research, Journal of Public Health, Volume 47, Issue 4, December 2025, Pages e616–e639, https://doi.org/10.1093/pubmed/fdaf084
*Pelton, L., and M. Knihtila. "Experience-Based Co-Design of health care services implementation guide." Institute for Healthcare Improvement, Boston, Massachusetts (2018).

Response: Thank you for these suggestions. We have read through the papers, and although they are very interesting, we have concluded that there are others that are more suitable for our study. We have therefore included references to the following studies to describe the principles of the co-design process in our study (page 6).

27. Bird M, McGillion M, Chambers EM, Dix J, Fajardo CJ, Gilmour M, et al. A generative co-design framework for healthcare innovation: development and application of an end-user engagement framework. Research Involvement and Engagement. 2021;7(1):12

28. Vargas C, Longworth GR, Zorbas C, Needham C, Ugalde A, Allender S. Establishing fundamental co-design principles and strategies for public health research practice. CoDesign. 2026:1-22.

29. Gustavsen B. Theory and Practice: The Mediation Discourse. In: Reason P, Bradbury H, editors. Handbook of Action Research. First ed: SAGE; 2001. p. 17-26.

3. Co-design/process for implementation from synthesising guidelines are recommended as follows:
• Engage stakeholders early (problem definition stage)
• Be explicit about roles and decision-making power
• Use multiple participatory methods
• Ensure ongoing iteration and feedback loops
• Invest in facilitation and relationship-building
• Evaluate both:
o outcomes
o quality of participation

Reply: Thank you for this overview, it is an helpful reminder. We have specified:
1) how stakeholders will be engaged from an early phase (pages 7, 12);
2) explicitly described the roles in terms of who will be responsible for analyses and decision making (Abstract/Strengths and limitations, page 9);
3) how we will use different participatory methods in terms of focus group interviews, in-depth interviews, dialogue conferences and discussions (see page 12 and Table 2 for a summary);
4) how iteration and feedback loops are included (pages 9, 11);
5) how the project will invest in facilitation and relationship-building (page 9);
6) a description on how both outcome and quality of participation will be assessed (Abstract/ Strengths and limitations, Table 2).

1) Page 7: *The services and stakeholders participating in the NorStep Colloaborate study have been recruited from the primary care services participating in the ongoing NorStep RCT study. Thirteen of the 21 services that participated in the RCT study in Fall 2024 were invited, based on their size (the largest services were selected), geography (services that were geographically and administratively situated in proximity to other services in the study were prioritized) and characteristics of their corresponding CAMHS (they were required to provide TF-CBT).*

1) Page 12: *The idea and research questions for this study is based on feedback from therapists and families participating in the NorStep feasibility study (16). Stakeholders (leaders and therapists) and children and caregivers with experience of being stepped up will be involved in this study from phase I.*

2) Abstract/Strenghts and limitations*: Due to limitations of stakeholder's time and resources, stakeholders will not be included as equal partners in the analysing phases, but an iterative strategy will be used to ensure stakeholder feedback during the whole process of development and evaluation of the model.*

2) Page 9: *After completion of the four dialogue conferences, the researchers will perform inductive thematic analysis (32) to interpret and systematize the contributions from the dialogue conferences.*

3) Page 12: *Leaders and therapists will take part in focus groups, dialogue conferences and iterative feedback loops on model drafts to co-design and refine the collaboration model.* (...) *Children and caregivers who have been stepped up from primary to secondary care in the NorStep RCT will be interviewed about their experiences of this transition, and their priorities and perceived needs will inform the development of an effective model for collaboration between primary and secondary health care levels.*

4) Page 9: *This analytical process will produce a proposed collaboration model that can be feasible across the pilot teams. A draft of the model will be sent to each participating service for review and feedback to prepare for a final meeting. Here, two representatives from each of the services included in this study will participate and agree upon a collaboration model that seems both feasible and effective.*

4) Page 11: *Finally, the results from the interviews will be summarized and presented to the pilot teams for their feedback. Based on the results, the collaboration model will be adjusted and consolidated.*

5) Page 9: *Throughout the day there will be multiple coffee breaks and a long lunch to facilitate relationship building among stakeholders in the different services, and all practicalities regarding the arrangement of the conferences will be handled by the research group.*

6) Abstract/ Strengths and Limitation: *The collaboration model will be evaluated both in terms of quality of participation (stakeholder involvement and to what degree the proposed actions were actually implemented) and outcome of the model (what are therapists', leaders', parents' and children's experiences with the collaboration after the model has been implemented).*

4. In the current protocol, the outcomes have not defined for children outcomes and quality of participation.
Response: Thank you for noticing this. We have now specified the topics of interests and questions to be addressed in the interviews in Table 2 (pages 11-12), including a specification of whether interviews will address quality of participation or an outcome.

Other minor comments:
1. The co-design include interview; it is not specified the interview method is semi-structure or in depth personal. Interview method needs to be described and justified.

Response: Thank you for this reminder, we have now specified the interview methods in Table 2 (pages 11-12).

2. The title include evaluation, while the method has not mentioned the outcomes or future

plan for evaluation in the co-designed model. Children's TB outcomes are not included in the evaluation.

Response: The primary aim of the NorStep Collaborate study is to learn more about how inter-agency collaboration around trauma-exposed children can be improved, and the main outcome is therefore on the quality of collaboration (in terms of implementation and perceived outcome, see Table 2) and not on child symptomatology. Outcomes in terms of improved health and functioning will therefore not be addressed in this study, but is however a main outcome in the NorStep RCT study (Clinical Trials registration no: NCT04073862). We have, mentioned that the lack of a control group is a limitation of this study as we will not be able to evaluate the effectiveness of the collaboration model (Abstract/Strengths and limitations).

Abstract/ Strengths and limitations:

- This study does not include a randomized control condition, so conclusions on the effectiveness of the collaboration model cannot be drawn.
